# X-ray computed tomography imaging of a tumor with high sensitivity using gold nanoparticles conjugated to a cancer-specific antibody via polyethylene glycol chains on their surface

**DOI:** 10.1080/14686996.2016.1194167

**Published:** 2016-07-26

**Authors:** Tomohiko Nakagawa, Kohsuke Gonda, Takashi Kamei, Liman Cong, Yoh Hamada, Narufumi Kitamura, Hiroshi Tada, Takanori Ishida, Takuji Aimiya, Naoko Furusawa, Yasushi Nakano, Noriaki Ohuchi

**Affiliations:** ^a^ Department of Nano-Medical Science, Graduate School of Medicine, Tohoku University, Sendai, Japan; ^b^ Department of Medical Physics, Graduate School of Medicine, Tohoku University, Sendai, Japan; ^c^ Department of Advanced Surgical Science and Technology, Graduate School of Medicine, Tohoku University, Sendai, Japan; ^d^ Department of Surgical Oncology, Graduate School of Medicine, Tohoku University, Sendai, Japan; ^e^ Development Department 4, Business Development Division, Business Development Headquarters, Konica Minolta, Inc., Tokyo, Japan

**Keywords:** CT, contrast agent, gold, nanoparticle, tumor, 30 Bio-inspired and biomedical materials, 504 X-ray / Neutron diffraction and scattering

## Abstract

Contrast agents are often used to enhance the contrast of X-ray computed tomography (CT) imaging of tumors to improve diagnostic accuracy. However, because the iodine-based contrast agents currently used in hospitals are of low molecular weight, the agent is rapidly excreted from the kidney or moves to extravascular tissues through the capillary vessels, depending on its concentration gradient. This leads to nonspecific enhancement of contrast images for tissues. Here, we created gold (Au) nanoparticles as a new contrast agent to specifically image tumors with CT using an enhanced permeability and retention (EPR) effect. Au has a higher X-ray absorption coefficient than does iodine. Au nanoparticles were supported with polyethylene glycol (PEG) chains on their surface to increase the blood retention and were conjugated with a cancer-specific antibody via terminal PEG chains. The developed Au nanoparticles were injected into tumor-bearing mice, and the distribution of Au was examined with CT imaging, transmission electron microscopy, and elemental analysis using inductively coupled plasma optical emission spectrometry. The results show that specific localization of the developed Au nanoparticles in the tumor is affected by a slight difference in particle size and enhanced by the conjugation of a specific antibody against the tumor.

## Graphical abstract

**Figure:**
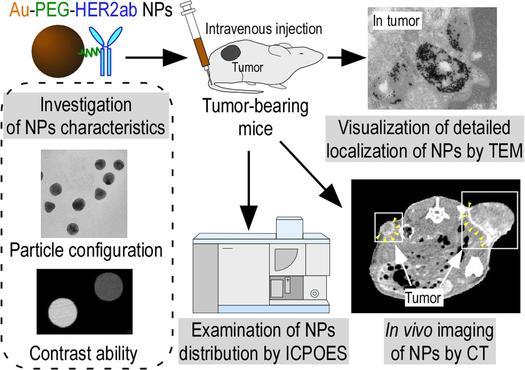

## 1. Introduction

X-ray computed tomography (CT) is commonly used for diagnostic imaging in clinical practice.[1] When evaluating a pathological lesion with CT imaging, it is necessary to differentiate between normal and diseased tissue sites. However, this is difficult when the contrast between the two sites is poor. In such a case, contrast agents are often used to enhance the contrast and improve the diagnostic accuracy. Iodine-based contrast agents, which have a higher X-ray absorption coefficient than living tissues, have been used for CT imaging in hospitals. However, there are two main problems with these clinical contrast agents. First, low molecular weight agents are currently preferred because they circulate through the bloodstream and are rapidly eliminated from the liver or kidney.[2] Because of the short circulation time, it is difficult to acquire multiple CT images or to determine the proper timing for CT imaging to obtain a clear image. Second, clinical contrast agents travel to extravascular tissues through capillary vessel walls, which depend on the concentration gradient. Therefore, the current contrast agents nonspecifically enhance all tissue, which limits tumor-specific imaging.

To overcome the limitations of the current contrast agents, blood pool agents are mainly used for developing magnetic resonance angiography contrast agents.[3,4] Blood pool agents differ from other magnetic resonance imaging contrast agents because they have a high molecular weight. Blood pool agents do not spread through the vascular epithelium because of their large size; as a result, they remain in the vascular system for a longer time period. Various nanoparticles have also recently been created as CT contrast agents for using CT imaging with nanotechnology.[5,6] Nanoparticles have different biodistributions in the body depending on their sizes and surface structures.[7,8] The changes in the biodistribution of nanoparticles are related to the structure of the capillary vessels. The structures of standard vessels consist of the tunica intima, tunica intermedia, and tunica adventitia layers. In contrast to the standard vessel structure, capillary vessels lack a tunica intermedia and tunica adventitia; instead, they consist of a monolayer of tunica intima or endothelium.[9,10] The endothelium monolayer permits the diffusion of oxygen, nutrients, and metabolism waste products from tissues to blood or blood to tissues. From a morphological viewpoint, the endothelium is divided into continuous and discontinuous types.[10] The difference between these types affects the vascular permeability. In the capillary vessels of normal tissues, the continuous endothelium is the main structure, and the endothelial cells are tightly arranged. Low molecular weight substances, such as the current contrast agents, can pass through the continuous endothelium. However, substances larger than several nm barely pass through continuous endothelium. In addition, there are discontinuous capillaries in the liver and spleen; these contain endothelial cells with clustered holes, called fenestra, that have diameters of approximately 60–180 nm. Therefore, nanoparticles less than 60 to 180 nm leak out of the fenestral structure.[11]

For tumor vessels, an enhanced permeation and retention (EPR) effect [12–14] has recently drawn attention as advantageous for nanoparticle delivery to the tumor. The EPR effect is a characteristic phenomenon for tumor tissues. The tumor induces the growth of neovascular vessels to increase nutrition and oxygen intake and promote growth. Tumor neovascular vessels differ from normal vessels in their anatomical structure. The tumor vessels are irregularly shaped, dilated, leaky and fragile. As a result, nanoparticles that are less than 150 nm can pass through the tumor vessel walls even though they cannot pass through the vessel walls of normal tissue. In addition, nanoparticles delivered to the tumors that have immature lymph network systems are difficult to eliminate through lymph vessels. This EPR effect is expected when developing a drug delivery system (DDS) and imaging tumors.

To date, some inorganic, nanoparticle-based CT contrast agents have been developed.[15,16] One of these is polymer-coated bismuth sulfide (Bi_2_S_3_). Bi_2_S_3_ shows higher X-ray absorption than iodine.[16] However, as it is difficult to control the size and shape of this agent, it is not suitable for clinical application. Here, we prepared Au nanoparticles as a CT contrast agent. Au has been applied in many areas of medicine, including incorporation into biosensors, DDS, optical imaging, and tumor radiotherapy.[17–19] The X-ray absorption coefficient increases in proportion to the atomic number. Au has a higher atomic number than iodine (Au, 79; I, 53) as well as a higher absorption coefficient (Au, 5.16 cm^2^ g^−1^ at 100 keV; I, 1.94 cm^2^g^−1^ at 100 keV). However, Au nanoparticles are unstable under physiological conditions and they easily aggregate. This aggregation inhibits the EPR effect and leads to vessel embolism. To enhance the EPR effect, sufficient blood retention of nanoparticles is essential [14,20,21] because the EPR effect is gradual, and the nanoparticle levels in tumors slowly increase over time. The nanoparticle surface is often modified with polyethylene glycol (PEG) chains to increase the dispersibility and blood retention of nanoparticles because PEG prevents plasma proteins from adsorbing onto the surface of the nanoparticles. When nanoparticles that lack special surface modification are exogenously administered into blood *in vivo*, plasma proteins easily adsorb onto the nanoparticle surface. Because adsorption prompts the phagocytosis of nanoparticles by macrophages (called the opsonin effect), plasma protein-adsorbed nanoparticles rapidly internalize into macrophages in the liver or spleen after leaking out of the fenestral structure. As a result, there is rapid clearance from the blood due to the uptake in the reticuloendothelial tissues of the liver and spleen.[22–24]

In this study, we prepared 30- and 15-nm Au nanoparticles carrying PEG chains on their surfaces (Au-PEG). Although there was only a slight difference between the Au-PEG sizes, 15-nm Au-PEG was more specifically localized into cancer tissues of tumor-bearing mice than 30-nm Au-PEG via the EPR effect. Human epidermal growth factor 2 (HER2) is a specific tumor biomarker in breast cancer. [25] We conjugated Au-PEG with the anti-HER2 antibody via terminal PEG chains (Au-PEG-HER2ab). Tumor-bearing mice were prepared using cultured cancer cells expressing HER2, and Au-PEG-HER2ab was injected into the mice. The nanoparticle distribution obtained by elemental analysis with inductively coupled plasma optical emission spectrometry (ICPOES) demonstrated the specific localization of Au-PEG when Au-PEG was bound to the anti-HER2 antibody.

## 2. Materials and methods

### 2.1. Syntheses of Au-PEG and Au-PEG-HER2ab nanoparticles

To prepare 30-nm Au-PEG,[26] 82.8 mg of chloroauric acid (Strem Chemicals Inc., Newburyport, MA, USA) was dissolved in 194 ml ultrapure water and heated to a boil. The chloroauric acid solution was vigorously stirred, and 20 ml of 39 mM sodium citrate (Wako, Osaka, Japan) was added to the solution under constant stirring. The sample was then boiled for 30 min. This sample solution included 30-nm Au nanoparticles. After cooling the sample solution to 25°C, 82.8 mg of HS-PEG-COOH (thiol carboxylic PEG, MW 5000, Nanocos Inc., New York, NY, USA) was added to the sample and incubated at 25°C for 12 h while stirring constantly to support PEG chains to the surface of 30-nm Au nanoparticles. After incubation, the sample was centrifuged at 22,000 × *g* for 40 min, and precipitates of Au-PEG nanoparticles were then suspended in phosphate buffered saline (PBS). These centrifugation and suspending processes were repeated three times to wash the samples. Finally, Au-PEG nanoparticles were resuspended in PBS.

To prepare 15-nm Au-PEG,[27] 99.4 mg of chloroauric acid was dissolved in 233 ml ultrapure water and heated to a boil. The chloroauric acid solution was vigorously stirred, and 28 ml of 39 mM sodium citrate was added to the solution under constant stirring. The sample was boiled for 30 min. This sample solution included 15-nm Au nanoparticles. After cooling the sample solution to 25°C, 99.4 mg of HS-PEG-COOH was added to the sample and incubated at 25°C for 12 h while stirring constantly to support PEG chains to the surface of 15-nm Au nanoparticles. After incubation, the sample was centrifuged at 22,000 × *g* for 40 min, and precipitates of Au-PEG nanoparticles were then suspended in PBS. These centrifugation and suspending processes were repeated three times to wash the samples. Finally, Au-PEG nanoparticles were resuspended in PBS.

To prepare 30- and 15-nm Au-PEG-HER2ab, 0.3 mg of EDC (1-ethyl-3-(3-dimethylaminopropyl)carbodiimide hydrochloride) (Thermo Fisher Scientific Inc., Waltham, MA, USA), 0.3 mg of sulfo-NHS (N-hydroxysulfosuccinimide), and 800 μl of PBS were added to 200 μl of 30- or 15-nm Au-PEG in PBS and constantly stirred for 30 min at 25°C. The sample was centrifuged at 22,000 × *g* for 40 min, and precipitates were then resuspended in PBS. Then, 20 μl of 20 mg ml^−1^ trastuzumab,[25] which is a monoclonal anti-HER2 antibody and HER2-target anticancer drug, were added to the sample and constantly stirred for 3 h at 25°C. The sample was centrifuged at 22,000 × *g* for 40 min, and precipitates of 30- and 15-nm Au-PEG-HER2ab nanoparticles were then suspended in PBS. These centrifugation and suspending processes were repeated three times to wash the samples. Finally, 30- and 15-nm Au-PEG-HER2ab nanoparticles were resuspended in PBS.

### 2.2. Experimental animals

We used female nude, 5–7 weeks old, from Charles River Laboratories Japan to develop the animal models. To create tumor-bearing mice, nude mice were subcutaneously injected with KPL-4 cells (2 × 10^6^), a human breast cancer cell line.[28] KPL-4 cells express HER2 at a high level. The KPL-4 cell line was provided by Dr J. Kurebayashi (Kawasaki Medical School, Japan). At 4–6 weeks after injection, the tumors were 1–2 cm in size. Necrosis was often observed inside the tumors due to rapid tumor proliferation. The mice were housed in a controlled environment, and food and water were provided ad libitum. The experimental protocols for using animals were approved by the University of Tohoku animal care committee.

### 2.3. Transmission electron microscopy (TEM) of Au nanoparticles

The Au-PEG suspension was directly dropped onto a collodion-coated copper grid (Nisshin EM, Tokyo, Japan), and images were observed using TEM (H-7600, Hitachi Science Systems, Tokyo, Japan). TEM was performed at an 80–100 kV accelerating voltage. We measured 100 Au nanoparticles to assess the average size and standard deviation. The 30-nm and 15-nm Au-PEG nanoparticles in solution did not aggregate for several months.

### 2.4. CT imaging equipment

CT imaging was performed by a micro-CT scanner (LCT-200 ALOK, Hitachi, Tokyo, Japan). The CT system can minimize motion artifacts when scanning living mice by using inhalation anesthesia. CT images were acquired at 50 kVp and 0.5 mA. The CT image size was 96 × 96 pixels, and the slice thickness was 384 nm. To evaluate enhancement by the contrast agent, the quantification of CT signals was expressed in Hounsfield units (HUs). CT data were analyzed using HUs in a region of interest.

### 2.5. CT imaging of Au nanoparticles

Using an *in vitro* study to evaluate the CT contrast value of Au-based nanoparticles, we measured the relationship between the Au concentration and the CT value and compared the two values with iopamiron 300 (300 mg of iodine per millimeter, 2.36 mol l^−1^, Bayer Schering Pharma, Leverkusen, Germany), a clinically used contrast agent. Each sample was placed in an approximately 1 cm^3^ thin plastic tube to measure the CT value. The CT values for each concentration of the Au-based nanoparticles or iopamiron were measured in a region of interest as HUs.

In the *in vivo* study, the mice were anesthetized and scanned at the baseline. Then, 200 μl of 0.2 M 30-nm Au-PEG, Au-PEG-HER2ab, 15-nm Au-PEG or Au-PEG-HER2ab was injected into the tail vein. The mean CT value was determined by setting the regions of interest.

### 2.6. Contrast effect of each organ after the injection of Au nanoparticles

The CT images were obtained before the injection of nanoparticles as well as 5 min and 3, 6, 12, 24, 48, and 72 h after injection. We measured the HUs of the regions of interest for CT images at each time point. The heart, vena cava, tumor, liver and spleen were observed by CT. To enhance the CT image analysis quantification, we focused on the tumor border, which included tumor vessels and the tumor center, which included a necrotic area. Then, we measured the CT values of these tumor regions.

### 2.7. TEM examination of organs after intravenous injection

We euthanized the mice 72 h after the intravenous injection of Au-based nanoparticles and removed their tumors. We then performed a histological examination by TEM. Tissue specimens were fixed and immobilized in 2% glutaraldehyde buffered with 0.1 M sodium cacodylate buffer solution (pH7.4). The tumor tissues were washed three times with 0.1 M sodium cacodylate buffer (pH 7.4), postfixed for 120 min in a solution of 1% OsO_4_ water, and dehydrated in 50% to 100% ethanol. Propylene oxide was used as a transitional solvent. The tissue was infiltrated overnight in a 1:1 mixture of Epon-Araldite and propylene oxide. The next day, the 1:1 mixture of Epon-Araldite and propylene oxide was removed and replaced with 100% Epon-Araldite. The tissue specimens were infiltrated with resin for 8 h and polymerized for 48 h at 60°C. The tissue was embedded in a resin, cut into 60-nm thick sections by ultramicrotome, and observed by TEM.

### 2.8. Biodistribution of gold by elemental analysis using ICPOES

The brain, liver, spleen, kidney, and tumor tissues were excised from the mice 72 h after the injection of 30- or 15-nm Au-PEG or 30- or 15-nm Au-PEG-HER2ab. The tissues were mixed with 2% SDS at 2× volume (ml) compared to the tissue weight (mg) and then treated with an ultrasonic homogenizer (Model UT-50, SMT Co., Ltd, Tokyo, Japan). The urine samples were mixed with 2% sodium dodecyl sulfate (SDS) at 2× volume (ml) compared to urine (ml). The elemental analysis of each sample solution was performed by ICPOES (SPS3520UV, Hitachi High-Technologies, Tokyo, Japan) to determine the gold level (mol/organ) in each organ (brain, liver, spleen, kidney, and tumor) and in the urine. A statistical analysis was performed. The differences in the ICPOES data were analyzed using the t-test. *P*-values < 0.05 were considered statistically significant.

## 3. Results

### 3.1. Preparation and characterization of Au-PEG nanoparticles

**Figure 1.**
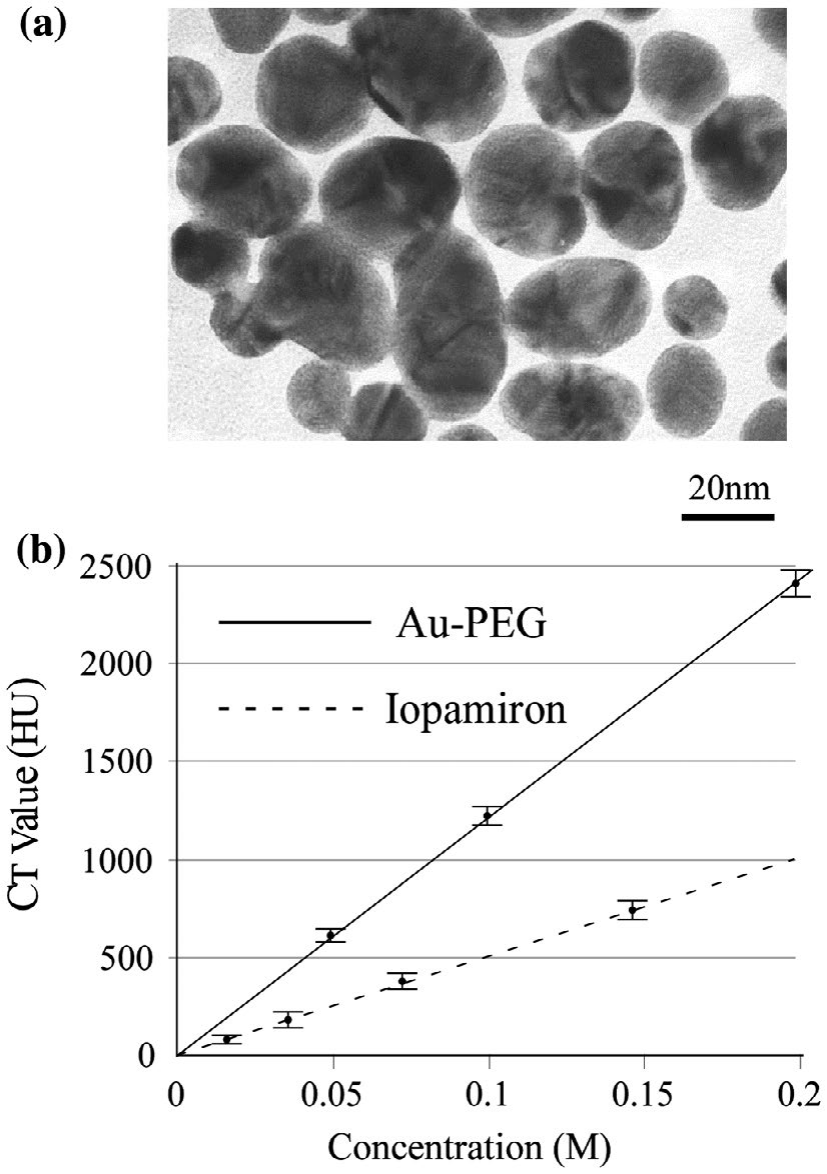
Properties of 30-nm Au-PEG. (a) TEM image of 30-nm Au-PEG. (b) Comparison of the contrast efficiency of 30-nm Au-PEG with iopamiron. There was a proportional relationship between the concentration and the CT value of Au-PEG. The CT value of Au-PEG was approximately twofold greater than that of the clinically used iodine agent at the same concentration. Error bars represent the standard error of the mean (SEM).

The EPR effect is effective for nanoparticles that are smaller than 150 nm.[29–31] We first created 30-nm Au-PEG nanoparticles as a contrast agent for X-ray CT imaging. This nanoparticle size was thought to be suitable for the EPR effect and would allow for easy tumor delivery. Figure 1(a) shows the TEM image of a 30-nm Au-PEG. The average size of the 30-nm Au-PEG was 30 ± 9 nm (mean ± standard deviation, SD). The Au nanoparticle solution was a deep red color as observed in a test tube.

Next, we investigated the contrast efficiency of 30-nm Au-PEG using CT imaging. There was a proportional relationship between the concentration and CT value of the Au-PEG nanoparticles (Figure 1(b)). When we compared the CT value of 30-nm Au-PEG nanoparticles with that of iopamiron, an iodine-based contrast agent used in clinical practice, the Au-PEG nanoparticles had CT values that were approximately twofold greater than the same concentration of iopamiron. This result shows that Au-PEG nanoparticles have significant potential as a CT imaging contrast agent.

### 3.2. Contrast effect of each organ in mice after injection of Au-PEG nanoparticles

**Figure 2.**
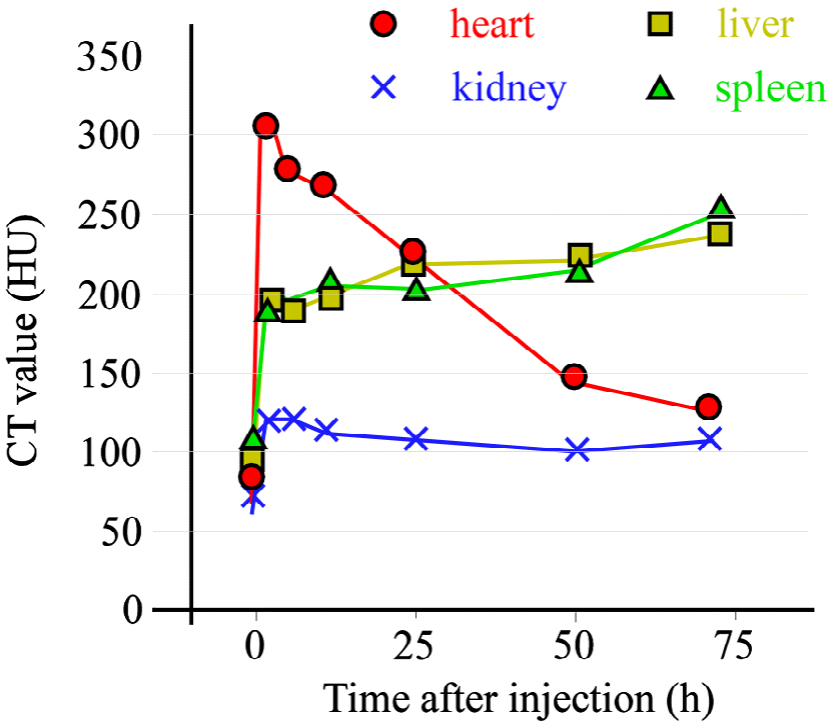
Change in CT value of each organ before and time after injection (h) with Au-PEG in tumor-bearing mice. The contrast effect of the heart rapidly increased and was retained for a long time. The effect gradually decreased. By contrast, the contrast effect of the liver and spleen gradually increased. In the kidney, there were no observable changes in CT value.

**Figure 3.**
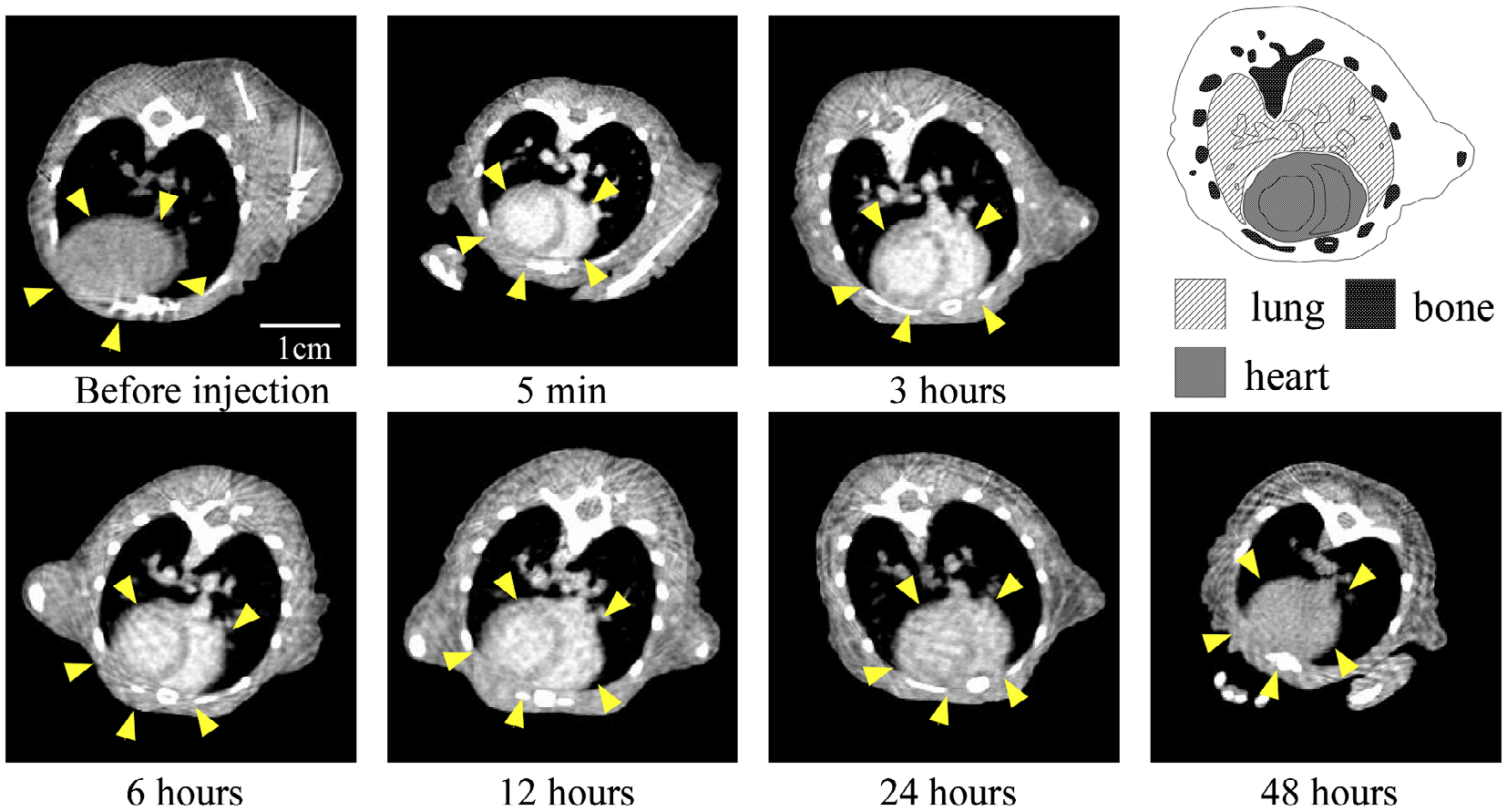
Change in CT value of a heart slice before and after injection of 30-nm Au-PEG. Yellow arrowheads indicate the region of the heart.

To examine the contrast efficiency of 30-nm Au-PEG by CT imaging, the Au-PEG nanoparticles were injected into mice, and the CT value of each organ (heart, liver, and spleen) was measured over time (Figure 2). First, we evaluated the change in the CT value in the heart. The CT value of the heart showed the retention of contrast agents in the blood. After injecting the mice with 30-nm Au-PEG, the contrast effects of the heart and vena cava rapidly increased and were maintained for a long time. The effect gradually decreased with time (Figures 2 and 3). The half-life of 30-nm Au-PEG in blood was 24 h (Figure 2). Although some contrast agents based on Au have been reported, this half-life was longer than for previously developed Au contrast agents [32]. The long retention in blood is important for obtaining an effective EPR effect because accumulation of contrast agents in the tumor occurs gradually. Therefore, the Au nanoparticles in this study were expected to be effective for tumor imaging.

**Figure 4.**
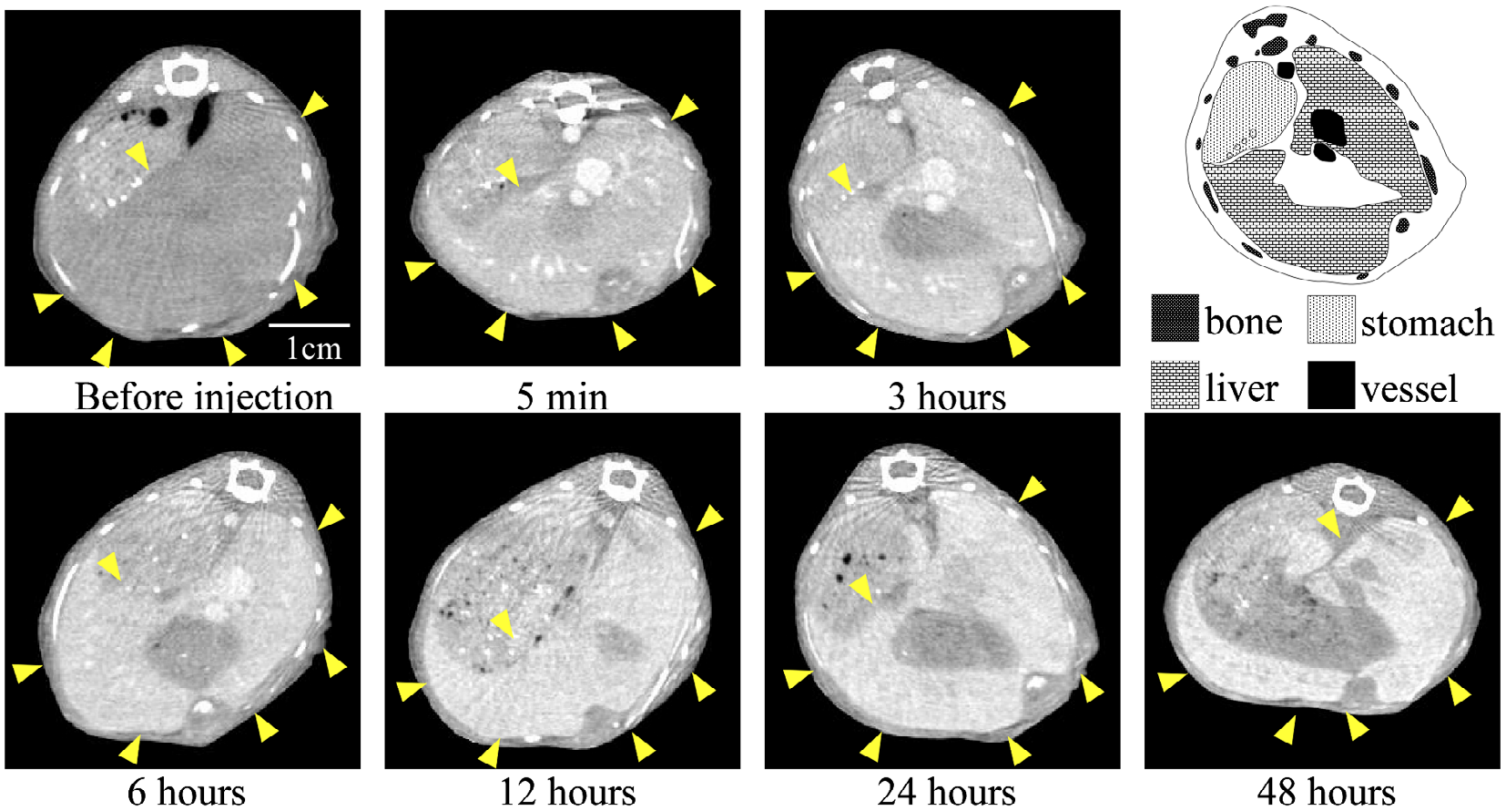
Change in CT value of a liver slice before and after injection of 30-nm Au-PEG. Yellow arrowheads indicate the region of the liver.

**Figure 5.**
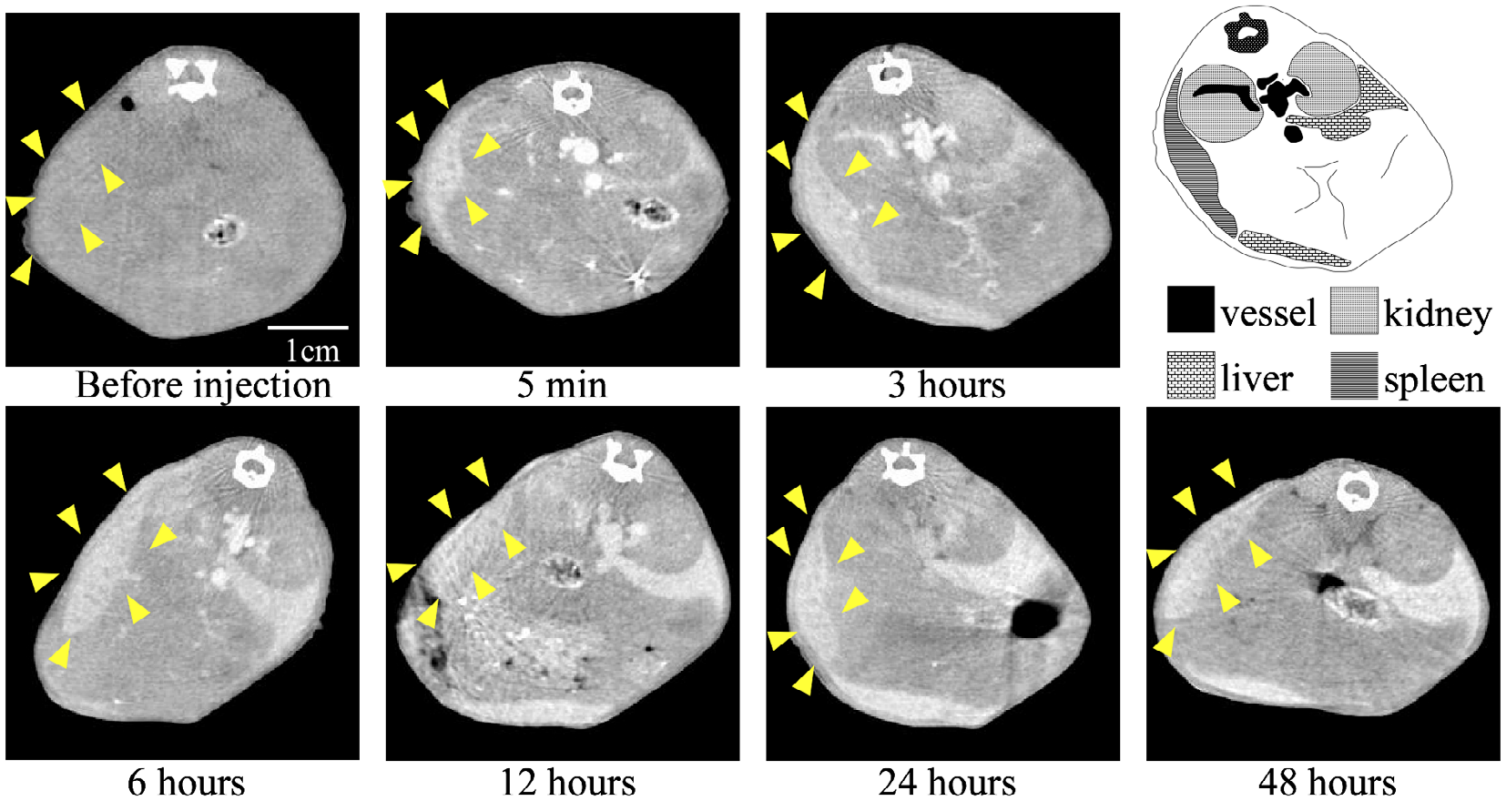
Change in CT value of a spleen slice before and after injection of 30-nm Au-PEG. Yellow arrowheads show the region of the spleen.

Next, we examined the changes in the CT values within reticuloendothelial tissues, including the liver and spleen. As the endothelial cells in the tissues have clustered holes, or fenestra, with diameters of approximately 60–180 nm, nanoparticles injected into the body are rapidly drawn into these structures. Therefore, the uptake of nanoparticles in the liver or spleen is a significant challenge to the delivery of nanoparticles into the target organ. Nanoparticle uptake into the liver or spleen often occurs within a few minutes.[24,27] Although the 30-nm Au-PEG also accumulated in the liver (Figures 2 and 4) and spleen (Figures 2 and 5), the nanoparticle uptake was not faster than for previously described contrast agents.[33] Because PEG chains on the surface of Au-PEG effectively inhibit plasma proteins from easily adsorbing onto Au-PEG, the retention of Au-PEG in blood was considered satisfactory. These results suggest that 30-nm Au-PEG is well-suited as a contrast agent.

### 3.3. Distribution of Au-PEG nanoparticles in murine tumors

**Figure 6.**
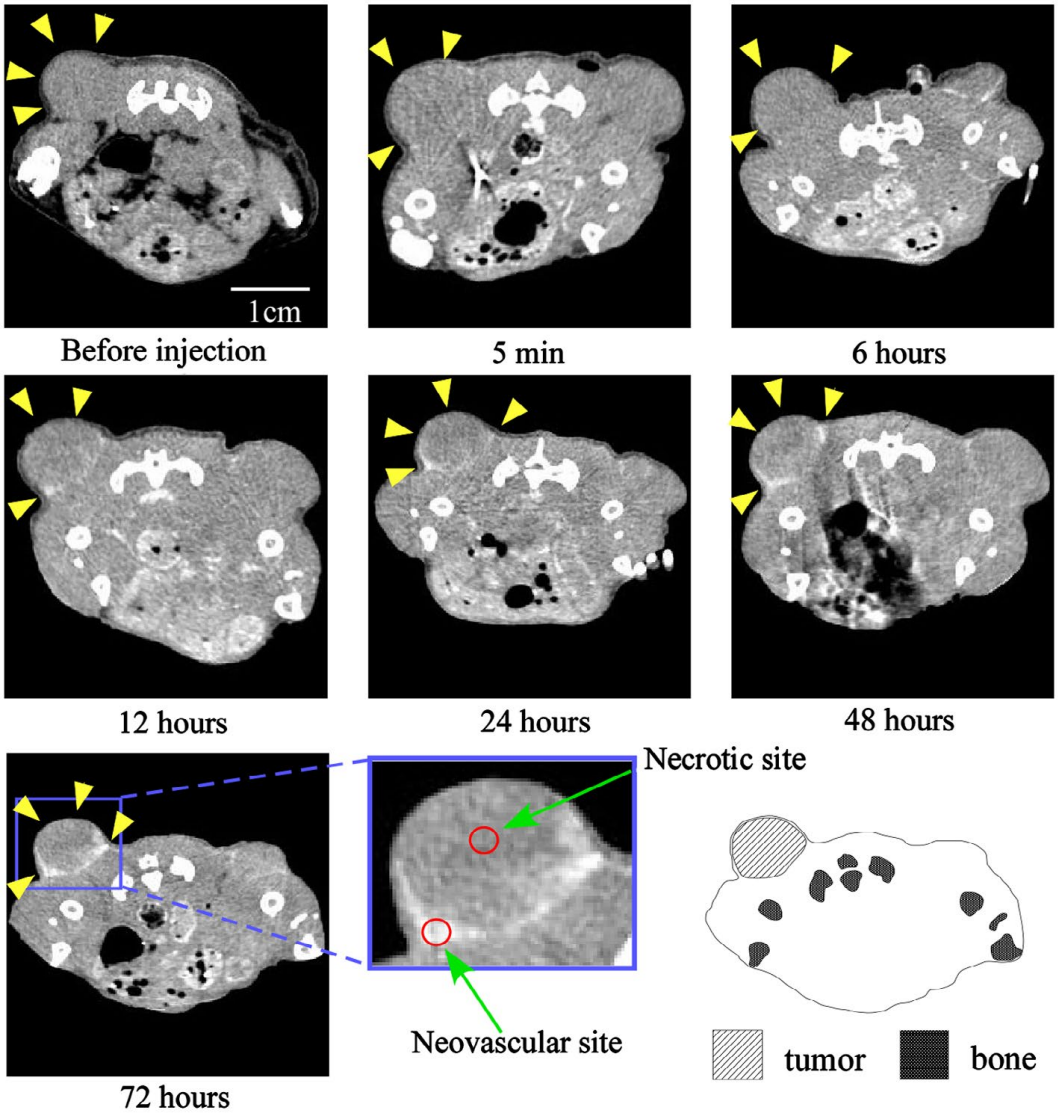
Contrast effect within a tumor after the injection of 30-nm Au-PEG. The tumor signal was greatly enhanced, and the tumor border was clearly imaged. Yellow arrowheads demonstrate the tumor region. The CT values of the regions of interest (ROIs), which are shown in red circles, were measured. The mean CT values of the necrotic site (tumor center) and neovascular site (tumor border) were 63 ± 12 HU and 235 ± 22. HU, respectively.

Because we succeeded in preparing an Au-PEG with good retention in blood, it was expected that Au-PEG would have a good EPR effect as well. The 30-nm Au-PEG nanoparticles were injected into tumor-bearing mice and imaged over time. The tumors gradually brightened after the injection of 30-nm Au-PEG (Figure 6). In particular, the tumor border was imaged clearly (Figure 6) because the outside layer of the tumor had many neovascular vessels, which is where the EPR effect tends to occur. By contrast, the signal inside the tumor was weak because the inside of the tumor has necrosis and neovascular vessels shrink with tumor growth.

**Figure 7.**
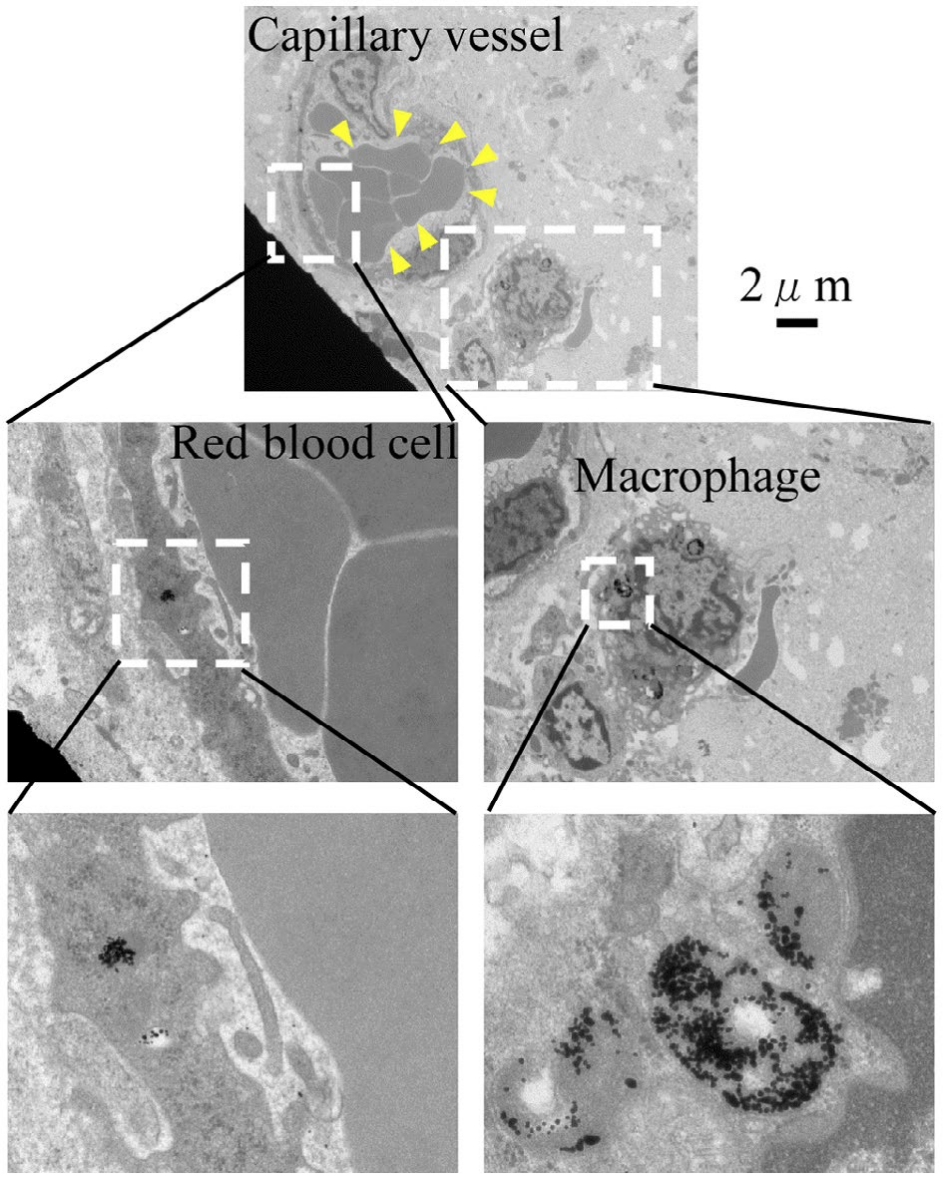
TEM examination of a tumor after injection of 30-nm Au-PEG nanoparticles. The nanoparticles were observed in the capillary vessel wall and were ingested by phagocytes around the capillary. Yellow arrowheads show the red blood cells in the capillary vessels of the tumor.

To demonstrate that Au-PEG nanoparticles were delivered to the tumor via the EPR effect, ultrathin sections of the tumor were obtained and observed Au-PEG accumulation was evaluated with TEM. Au-PEG nanoparticles were observed in the capillary vessel wall; they had been ingested by phagocytes in the capillaries around the tumor (Figure 7), suggesting that the EPR effect of Au-PEG was effective.

### 3.4. Examination of the Au-PEG biodistribution by elemental analysis using ICPOES

**Figure 8.**
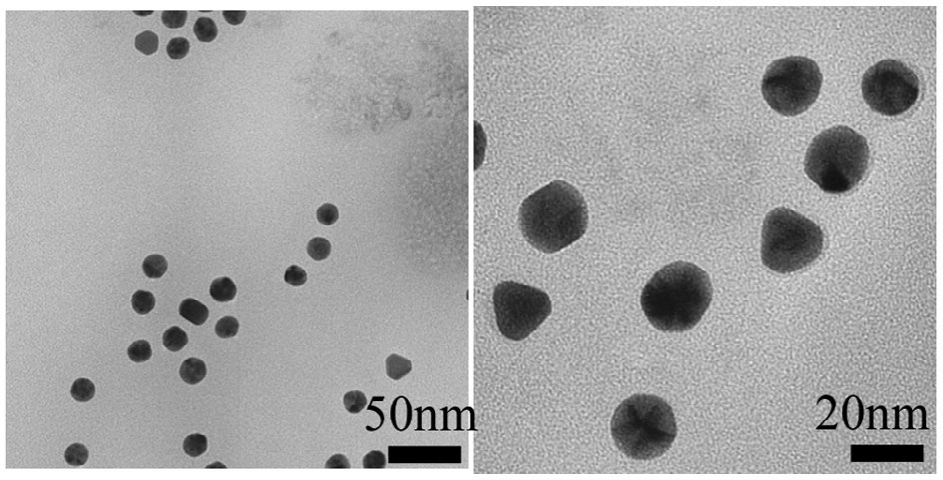
TEM image of 15-nm Au-PEG nanoparticles.

**Figure 9.**
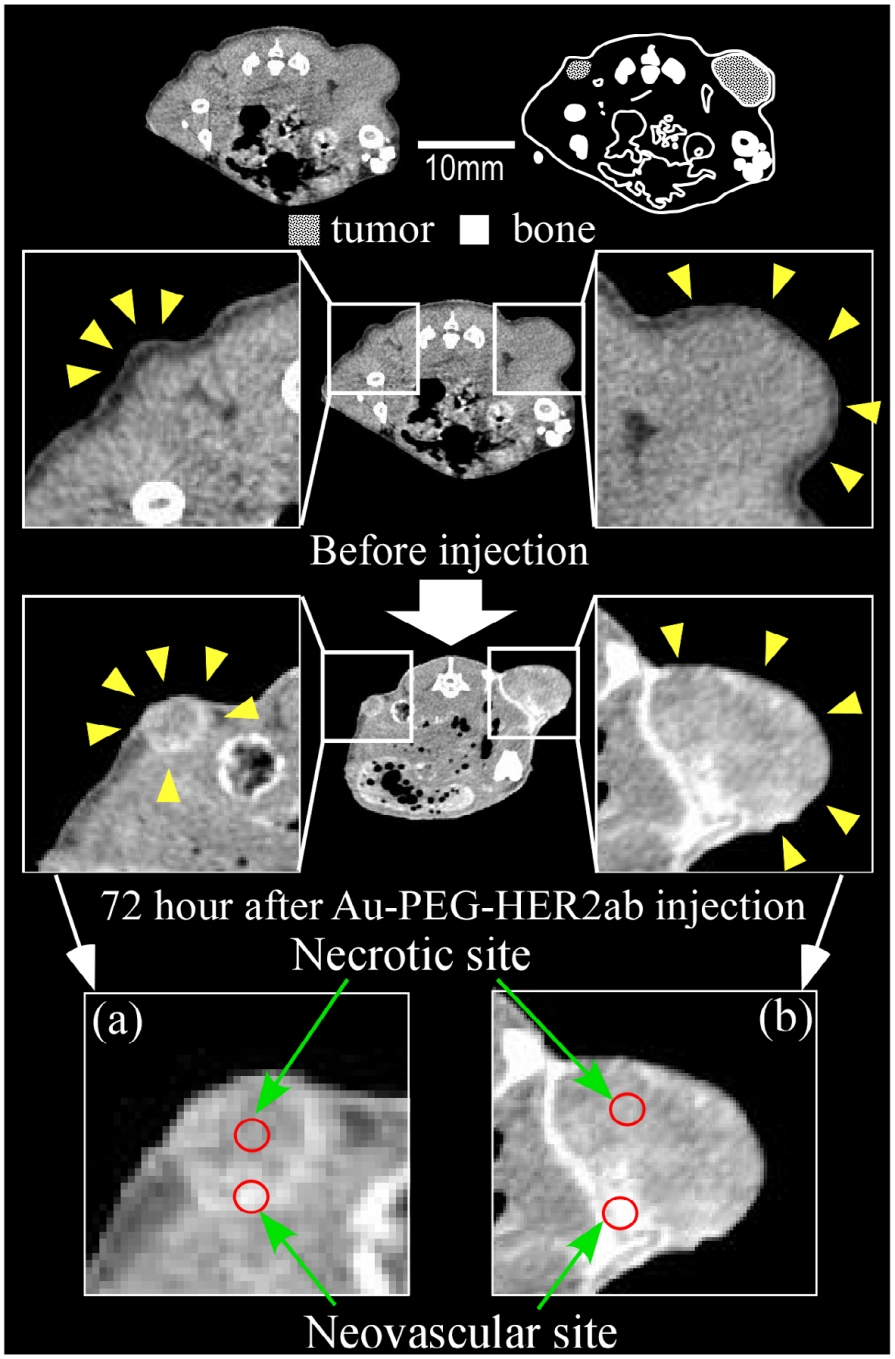
Contrast effect within a tumor after the injection of 15-nm Au-PEG nanoparticles. The Au-PEG nanoparticles enabled us to image a microtumor that was only a few millimeters in size in mice, while 30-nm Au-PEG did not. The right and left images show large tumors and microtumors, respectively. Yellow arrowheads demonstrate the tumor region. The CT values of the ROIs (red circles) were measured. The CT values of the necrotic and neovascular sites in (a) were 72 ± 14 HU and 244 ± 33 HU, respectively. The CT values of the necrotic and neovascular sites in (b) were 60 ± 15 HU and 280 ± 45 HU, respectively.

**Figure 10.**
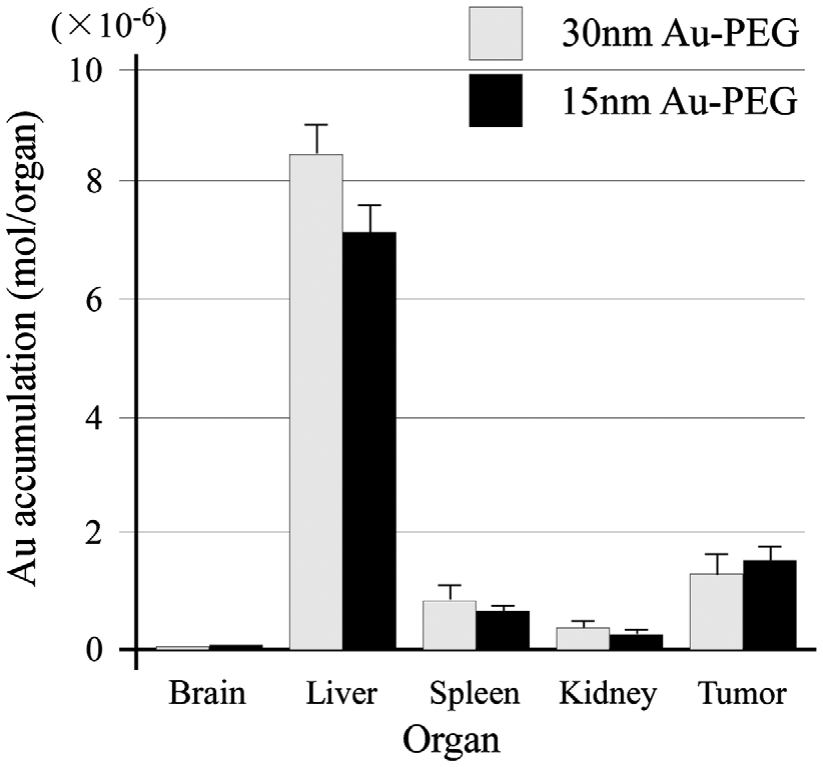
Measurement of the Au biodistribution using elemental analysis with ICPOES 72 h after the injection of 30- or 15-nm Au-PEG. The *y*-axis shows the Au accumulation (mol/organ 1 mg). The *x*-axis shows the distributions of 30- and 15-nm Au-PEG in the brain, liver, spleen, kidney, and tumor. The graph shows the average values for three mouse experiments. Error bars, SEM.

The EPR effect is thought to occur for nanoparticles smaller than 150 nm. To investigate whether a 30-nm Au-PEG is stable for effective delivery to the tumor via the EPR effect, we compared 30- and 15-nm Au-PEG samples. Figure 8 shows the TEM image for a 15-nm Au-PEG. The 15-nm particles were uniform in size with an average size of 15 ± 3 nm (mean ± SD). The 15-nm Au-PEG nanoparticle solution did not aggregate for several months, indicating it had high stability that was comparable to the 30-nm Au-PEG solution. We injected 15-nm Au-PEG into the tumor-bearing mice. The 15-nm Au-PEG sometimes enabled us to image a microtumor that was only a few millimeters in syngeneic mice (Figure 9), while the 30-nm Au-PEG did not. As tumors consist of heterogeneous cancer cells, unlike the liver and spleen, comparing the distributions of 30- and 15-nm Au-PEG in tumors with high accuracy by CT imaging is difficult. Therefore, we evaluated the distribution of 30- and 15-nm Au-PEG in tumors by elemental analysis using ICPOES because ICPOES offers superior sensitivity for measuring the Au element concentration compared to CT imaging. In control mice, which were not injected with Au, Au was not detected. In addition, Au was not detected in the urine or stool samples of mice injected with Au-based nanoparticles. We measured the gold levels (mol/organ) in each organ (brain, liver, spleen, kidney, and tumor) with ICPOES. For the mean gold level observed with ICPOES, the levels in the liver and spleen for the 15-nm Au-PEG were lower than for the 30-nm Au-PEG; conversely, the tumor level was higher for 15-nm Au-PEG than for 30-nm Au-PEG (Figure 10). However, there was not a statistically significant difference in the biodistributions of 15- and 30-nm Au-PEG.

### 3.5. Enhancement of the contrast effect in tumors by cancer-specific antibody-conjugated Au-PEG

**Figure 11.**
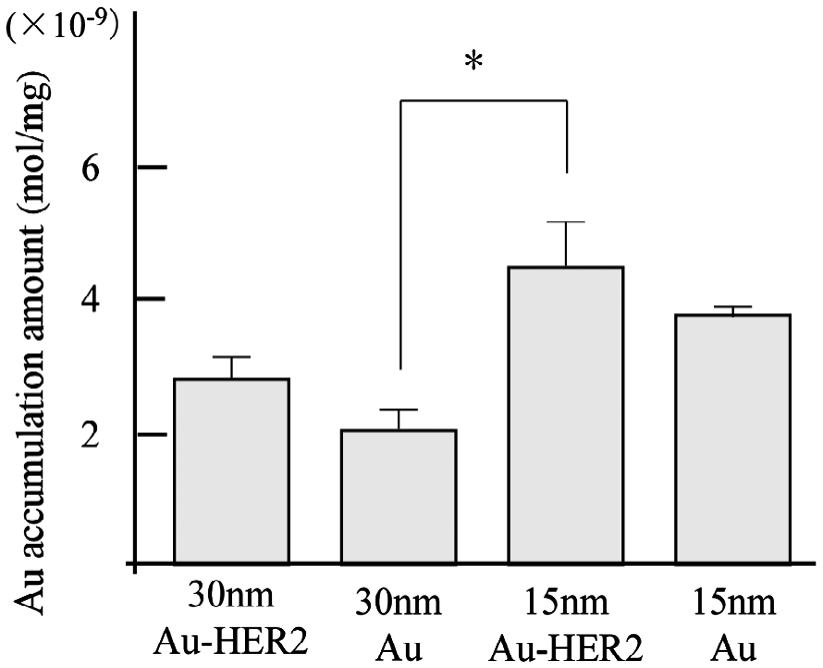
Measurement of Au accumulation in a tumor by elemental analysis using ICPOES when 15- or 30-nm Au-PEG-HER2ab or 15- or 30-nm Au-PEG nanoparticles were injected into tumor-bearing mice. The *y*-axis shows Au accumulation (μmol mg^−1^). On the *x*-axis, 30-nm Au-HER2, 30-nm Au, 15-nm Au-HER2, and 15-nm Au show 30-nm Au-PEG-HER2ab, 30-nm Au-PEG, 15-nm Au-PEG-HER2ab, and 15-nm Au-PEG, respectively. The graph shows the results in three mice experiments. Error bars, SEM. *Significant difference (*p* < 0.05).

To increase the level of contrast agent delivered to tumors, contrast agents were conjugated to cancer-specific antibodies, which effectively enhanced the tumor-targeting ability. In breast cancer, HER2 is a specific biomarker for tumor tissues. Trastuzumab is a humanized monoclonal anti-HER2 antibody and HER2-target anticancer drug used in clinical practice.[25] We conjugated Au-PEG with the anti-HER2 antibody via terminal PEG chains (Au-PEG-HER2ab). Tumor-bearing mice were prepared using cultured cancer cells expressing HER2, and 15- or 30-nm Au-PEG-HER2ab or 15- or 30-nm Au-PEG nanoparticles were then injected into the mice. The tumor distribution of each nanoparticle (Au mol / tumor 1 mg) was analyzed by elemental analysis using ICPOES, as shown in Figure 11. The 15-nm Au-PEG-HER2ab had the best contrast of the Au-based contrast agents in three murine experiments (Figure 11), suggesting that the localization of Au nanoparticles within a tumor is enhanced based on the nanoparticle size and tumor antibody conjugation.

## 4. Discussion

In this study, we prepared 30- and 15-nm Au-PEG nanoparticles that demonstrate twofold greater contrast for CT imaging compared to iopamiron, an iodine-based contrast agent used in clinical practice. As high contrast enhances the tumor detection, Au-based nanoparticles are expected to be useful for the early detection and accurate diagnosis of cancer. We succeeded in imaging microtumors that were only a few millimeters in size with the injection of 15-nm Au-PEG into tumor-bearing mice.

We could directly observe the EPR effect with 30-nm Au-PEG using TEM imaging of the tumor (Figure 7). Although many reports have demonstrated DDS against tumors via the EPR effect with appropriately sized nanoparticles, direct observation of the EPR effect by TEM imaging is rare. Therefore, the TEM findings in this study of Au-PEG nanoparticles within the tumor vessel wall, actively being ingested by phagocytes around the tumor vessel, provide valuable evidence for the EPR effect.

The tumor neovascular vessels differ from normal vessels in terms of their anatomical structure. Tumor vessels are rapidly formed by an unregulated system, which is in contrast to the regulated system used for normal vessel formation. This is thought to lead to an increase in the gaps between vascular endothelial cells in tumor vessels, and nanoparticles smaller than 150 nm can pass through the tumor vessel walls without passing through normal tissue vessel walls.

The CT values of the liver and spleen were gradually increased by the injection of Au-PEG nanoparticles. This indicates that there is gradual accumulation of Au-PEG in reticuloendothelial tissues in the liver and spleen. In these tissues, the endothelial cells of capillary vessels have clustered holes, or fenestra, on their walls with a diameter of approximately 60–180 nm. Therefore, Au-PEG nanoparticles, which are smaller than 60 nm, leak out of the capillary vessels through the fenestra, which contributes to the EPR effect in tumors. There are many macrophages, such as Kupffer cells, in reticuloendothelial tissues. Therefore, after Au-PEG leaks out of the vessels in reticuloendothelial tissues, macrophages in the tissues phagocytize Au-PEG. This is thought to contribute to the gradual increase in the CT values of Au-PEG in the liver and spleen. Au-PEG nanoparticles are decorated with surface PEG chains. As PEG can reasonably avoid rapid adsorption by plasma proteins, which otherwise induces rapid uptake into the liver and spleen, Au-PEG nanoparticles are only slowly and gradually incorporated into the liver and spleen. When the PEG surface modification of nanoparticles is insufficient, plasma proteins can easily adsorb onto the nanoparticles, which prompts macrophage-mediated phagocytosis. This is a major mechanism for removing foreign materials from the blood circulation and protecting the body. The Au nanoparticles in this study were present in the body for a long period of time. Therefore, it seems unlikely that Au nanoparticles are excreted from the body. We did not thoroughly examine the effect of Au remaining in the murine body for a long period of time. However, the mice remained in good physical condition throughout the study and maintained a sufficiently long lifespan. In future studies, we will examine the biodistribution of Au by elemental analysis and safety over different time periods using serum biochemical analysis.

In this study, to more precisely deliver Au-PEG nanoparticles to tumors, we conjugated Au-PEG with trastuzumab, an anti-HER2 antibody, on the surface via PEG chains. We injected Au-PEG-HER2ab into the tail vein of tumor-bearing mice. X-ray CT images showed that Au-PEG-HER2ab nanoparticles were delivered into the tumor in the same way as Au-PEG (data not shown). However, as tumors consist of heterogeneous cancer cells, unlike the liver and spleen, it is difficult to accurately compare the distribution of Au-PEG and Au-PEG-HER2ab in tumors using CT imaging. Elemental analysis using ICPOES demonstrated that the accumulation of 15-nm Au-PEG-HER2ab in tumors was the highest of the Au-based nanoparticles in this study. It is thought that 15-nm and 30-nm Au-PEG were delivered into the tumor by passive targeting, while 15-nm and 30-nm Au-PEG-HER2ab were delivered into the tumor by passive and active targeting due to the affinity of the HER2 antibody to tumor cells. For passive targeting with the EPR effect, Au accumulation of 15-nm Au-PEG into tumors was 1.86-fold greater than that of 30-nm Au-PEG. For both passive and active targeting, the Au accumulation of 30-nm Au-PEG-HER2ab into tumors was 1.35-fold greater than that of 30-nm Au-PEG, and the Au accumulation of 15-nm Au-PEG-HER2ab into tumors was only 1.19-fold greater than that of 15-nm Au-PEG. However, when the size effect was considered with passive and active targeting, the Au accumulation of 15-nm Au-PEG-HER2ab into the tumor was 2.2-fold greater than that of 30-nm Au-PEG (Figure 11, *p*-values < 0.05), suggesting that the size effect, passive targeting, and active targeting work together to effectively deliver nanoparticles. These results suggest that both limiting the nanoparticle size to less than 15 nm and modifying the surface of the nanoparticles with a cancer-specific antibody are effective for preparing effective tumor imaging contrast agents. Based on the findings of this study, it is expected that superior Au-based contrast agents will be available for clinical practice in the near future.

## Funding

This work was supported by a Grant-in-Aid for Scientific Research on Innovative Areas ‘Nanomedicine Molecular Science’ [23107009] of the Ministry of Education, Culture, Sports, Science, and Technology, Japan (K. Gonda), Japan Society for the Promotion of Science (JSPS) KAKENHI [grant number 16H05168 (K. Gonda) and 16K15572 (K. Gonda)], and a Grant-in-Aid for A3 Foresight Program from JSPS (K. Gonda and N. Ohuchi).

## Disclosure statement

No potential conflict of interest was reported by the authors.
